# Monthly Periscope

**Published:** 1853-09

**Authors:** 


### EDITORIAL DEPARTMENT.

*Monthly Periscope.*—In the Transactions of the College of Physicians of Philadelphia, is published a biographical memoir of the late Dr. Daniel Drake, by C. D. Meigs, M. D. We have read it with that deep interest, which is inseparable from watching a career distinguished by genius, and marked by vicissitudes. Dr. Drake left uncompleted his great task of describing the diseases of the West, their topography, and causes. One human life was too brief for this vast undertaking; and, though Dr. Drake had at his death, reached almost to the three score years and ten appointed unto man, he died with his labors uncompleted, his ambition unsatisfied, and his lofty hopes in a measure unrealized. Young men *must* die thus. Cut down in early manhood, the scholar must leave the world, unsatisfied with his accomplishments. And the mais youthful ambition remained with Dr. Drake to his latest moments. He *had* accomplished very much to give his name a claim upon posterity — he had *not* accomplished *all;* because his ambition soared beyond his strength, and the term of life allotted to him. He lived sixty-seven years; but we look upon his death with the same unsatisfied feeling that we have when thinking of Bichat, dying at the age of twenty-seven.

*Fractured Clavicle.* — Dr. Crawford, of McGill College, publishes in the Montreal Medical Chronicle, a description of a new adjuster for fractured clavicle. Among the multitude of new splints, and other apparatus offered, we may hope for escape from suits for malpractice. This adjuster consists of a truss-like, padded band of steel, passing round the body and beneath the axilla of the affected side, secured by buckles and straps. At its axillary portion, protrudes a steel fork, (likewise padded,) which embraces the arm. This makes the trunk of the body the *point d'appui.* A strap and buckle passing over the shoulder, prevents its slipping down. The arm is then suspended in a sling. It seems to us, that the peculiar difficulty in managing this fracture, is in the want of an immovable point of resistance, from which to support, and throw back the shoulder. This apparatus has the merit of simplicity, and fixes upon the walls of the chest, as the point of resistance. It promises well, but it is difficult to predicate its success.

The neat and convenient splint for fractures of the lower forearm, described in our original department, may be seen at Mathews' drug store, in this city.

*Translations of Ricord's 'Letters upon Syphilis.'*—The long agony is over. Simultaneously and with one accord, some half a dozen ambitious souls began, continued, and completed these translations. Philip Ricord has been duly canonized. He is beatifically displayed, surrounded by a glorious halo of chancres and phagadenic ulcerations, while a misty fountain of blenorrhagia showers its spray around him. We have been duly enlightened as to the fact, that syphilis is a self-protective disease; that it may not occur twice in the same individual; or at any rate, that a succession of inoculations will enable the whoremonger and adulterer to sin unharmed. Glorious conclusion!

We would say nothing to discourage any branch of medical research. Undoubtedly it is our duty to heal the infirmities of vice; but we confess to an inability to appreciate the utility of these apotheosized letters. Their great object seems to be, to prove that syphilitic disease grows harmless by repetition — an assertion which is not at all supported by the cases adduced; most immoral in tendency; and only sustained by the most turgid, and verbose style of argument, we have ever perused. We object strongly to the style and tone of these letters. From the uniformity of the several translations, we are led to believe that the original itself was almost unintelligible; and the pomp and diction which is thrown about them, is unfit for the consideration of any medical subject. A plain direct style is a great desideratum in medical writing; but there is an owlish dogmatism about these letters which we cannot reconcile with our ideas of the simplicity of "divine philosophy." Undoubtedly, M. Ricord is the first "syphilographist" now living, and his opinions are consequently entitled to much respect; but we think we can discover in these letters, the effect of that flattery, which has for a succession of years been so lavishly bestowed upon him, as well as an over anxiety to maintain his reputation by the production of something new.

In looking over our exchanges, we sometimes see changes in medical opinion indicated rather by the tone, than the expressed declarations of writers. Sundry unaccustomed words come into common use, and we see from such straws the direction of the prevalent wind. Lately, while no one deems it necessary to come out as the special advocate of the humoral pathology, it is still evident that solidism has had its day, and that by a silent yet effective agency, a change of doctrine has come about which is too important to pass unnoticed. A new classification of disease is being made. By a close insearch into ultimate causes, physicians have come to regard diseases widely separated in symptom as one in essential nature.

Thus under the head of zymotic disease, we have now enumerated not only the contagious exanthemata, but also diarrhœa, dysentery, cholera, the non-eruptive fevers, (intermittent, remittent, and congestive,) neuralgia, rheumatism, (though that may be considered as an eruptive fever,) and finally pleurisies and pneumonias. In all of these the modern notion is, that zymosis, or the increase of poison in the blood by a process analogous to fermentation, is the *fons et origo mali.*

Such a belief must necessarily make some change in therapeutics. Self-limitation becomes a word of meaning. We shall act no longer on the notion of the division of disease into two great departments—phlogistic and non-phlogistic. We shall see that diseases presenting fever as a symptom, or entirely without fever, may acknowledge the same cause, and require the same treatment. Standing behind this subject, enveloped in a most formidable obscurity, is the question of malarial influence—its unity or divisibility, and its capability of producing various diseases. While we believe that we are approximating to truth on this great question, we are still free to confess that in our own mind there is nothing approaching a conviction, and we find as yet no ground to rest upon. But we may reason, (Lord Bacon to the contrary notwithstanding,) from final causes.* A correct appreciation of the true cause of this class of disease is of the utmost importance, and we believe that as the consideration of final causes resulted in the Harveian discovery of the circulation, so here the consideration of the causes which conduce to epidemic and zymotic disease shall through much difficulty guide us to a true appreciation of blood poisons. By considering the known phenomena of disease, we may prove *analogically* the unknown causation, and at least by knowing what it behooves us to prove or disprove, we shall be able to establish our analogy by inductive reasoning.

So far as present knowledge goes, we have already demonstrated to us, a relationship between the diseases enumerated. Whether this relationship extends to causation we do not propose to consider, and the object of this argument is to trace those features which are common to all zymotic disease. In the contagious exanthems, all the symptoms go to prove the existence of

---

* My Lord Verulam says, "that final causes, like the vestal virgius, are devoted to the service of the Divinity, but like them too, are unfruitful, the consideration of them not leading to scientific improvement, and natural discoveries."

a blood poison which seeks an outlet by the skin or mucous membranes. Typhoid fever is not an exception to this rule, for the mucous ulcerations in the bowels are not irrationally regarded as conservative—a representative, as it were, of the variolous pustule on the skin.*

It is argued that in this class of disease there are three stages: 1st. The period of incubation, dating from the reception of the morbific ferment. 2d. The period of development, during which the poison creates the necessary pustulation, ulceration, or other outlet for its escape; and thirdly the period of elimination or decline. From this we draw the conclusion, that the cutaneous eruption, when there is one, is a symptom only to be checked at the risk of translation to the more vital organs, and that our management of those zymotics which relieve themselves, and eliminate their poison by discharges from the alimentary canal, should be such as to guide, rather than to cut short, the discharges. Hence in our opinion the efficacy of the saline purgative in dysentery, and of that treatment of diarrhœa and cholera, which attempts, and often successfully, the substitution of urinary and "bilious" discharges, for the serous evacuation peculiar to the disease. The success of the pure opium treatment, (which it is useless any longer to dispute,) is no contradiction of our hypothesis, though its *modus curandi* is different. In all zymotics death occurs from one of two contingencies: 1st. From the direct agency of the poison upon the nervous system overpowering it as if by narcosis. Instances of this are found in those cases where the system sinks under the first invasion of a fever—the retrocession of an eruption, or in those cases of cholera which die in collapse, without sufficient discharge to account for death, or which sink into asthenia, and perish seemingly from loss of nervous energy, also without remarkable discharges. Secondly, death may occur from exhaustion of the fluids, from the mere draught upon the circulation, as in dysentery, or cholera, with that profuse discharge which ordinarily accompanies it.

---

* It has recently been argued from the increasing prevalence of typhoid since general vaccination, that the divine law of compensation is at work equalizing the ravages of death. Population does not increase in any greater ratio since, than before the time of Jenner, and it is thought that the lessening frequency of variola finds its compensation in increasing typhoid, and that, (supposing a certain and limited amount of corruption to be generated in a nation of men,) the depurative agencies set up by the Deity to partially counterbalance the destructive processes, find elimination in typhoid, *because* they have lost the outlet afforded by the small-pox. In a word we must "by foreordination and decree," submit to one or the other. This is what may be called transcendental pathology.

Now opium acts in two ways to combat death. In the first class of cases, it sustains by its stimulus the spinal system under the shock of the poison. It directs to the surface, and equalizes the circulation, and while it has (aside from the perspiration it may induce) no agency in removing the poison from the system; it guides to a cure by giving endurance to the powers of life, and tempering to them the attack which would otherwise be insupportable. Leaving the cure entirely to the natural process of elimination, it still so guides and governs that process, as to conduce to recovery. The curative influence of iron in erysipelas, must also be referred to its sustentation of the nervous system.

In the second class, it checks and controls the process of elimination in such a manner, as to prevent that drain upon the circulation which is in the end more fatal than the poisonous cause itself.

One word about final causes. We have inverted in our argument the Baconian process, and have reasoned from the treatment to the disease. We have from a never-failing analogy, supposed that there must be some reason—some final cause—why the treatment by opium, by calomel, or by nothing at all, should in this class of disease have almost equal claims upon our confidence. We have endeavored to find this cause in the phenomena of the diseases under consideration. In doing this we have reasoned from a group of diseases, for the same reason that an argument drawn from a group of cases, is more reliable than one derived from a single instance.*

Our reasoning will apply *mutatis mutandis*, to all the diseases we have named. Look at their treatment, and you will find that the same principle governs them all. As a general rule, the process set up by nature, is the safest one for the elimination of the poison. Where this, however, is too harsh and exhausting, as in cholera, dysentery, rheumatism, and gout, it should be checked, and controlled by opium. In the periodical fevers, the poison being different, acts in a different way, and is best controlled by exalting the nervous system above its influence. This may be sometimes done by opium, and sometimes by quinine.

---

* If we were writing an "introductory lecture," we should not dare to depart from the time-honored custom, of exalting the perfections of the Baconian, or inductive philosophy. It is a stereotyped habit with the learned lecturers who annually publish, at the request of their classes, their neatly written introductions, to glorify Lord Bacon; but in the free and easy pages of our periscope, we may venture to depart from it, and express our belief, that reasoning by deductions, by synthesis, and analogy, are very honest ways of getting at the truth. If all that *claims* to be induction, is truly so, then it is the most fallacious of all reasoning.

In the treatment of erysipelas, scarlatina, rubeola, and variola, the same *a tergo* reasoning teaches us, that the natural habitat of the eliminating process being the skin, we should encourage the natural tendency toward that organ. If, however, its direction should be toward the nervous centres, through retention in the circulation, then we should sustain the nervous system. It seems that if the system is already saturated with one poison, it does not feel so readily the influence of another. A man very drunk, being bitten by a rattlesnake, (animal poisons are zymotic diseases,) was unharmed. Numerous instances are recently recorded, of the alcoholic poison being introduced into the system early enough to pre-occupy, or in quantity enough to overcome the reptile poison, and the usual fatal result was avoided. So, too, of cases of *cobra di capello* poisoning cured by the aromatic spirits of ammonia. It is thus evident, that the sustentation of the nervous system is the most important indication in zymotic disease, the system being usually able to sustain the elimination of the poison by the skin, or mucous membrane, without a fatal result.

*Veratrum Viride again.*—A friend, to whom we appealed for information as to the remedial properties of this drug, replies as follows in a private letter: "I used it in four cases of pneumonia; and in six or eight other cases. In one (the first) case, I liked its apparent effects, and thought it would sustain itself. The case was mild, and would have terminated favorably of itself I presume. But my expectations in the second case, faint as they were, were not realized, and tart. ant. et pot., with a blister, came to my relief; but the run was closer than I liked to a fatal result.

"I have not now the confidence in it that I have in colchicum, digitalis, or aconite; and neither of them are comparable to tart. ant. in my hands. It will not 'rise again' in my practice. I used it according to Dr. Norwood's method."

We are of the opinion that others will find it equally nugatory for good. Any new remedy, if really valuable, should be readily adopted, but our materia medica is already too cumbersome to admit articles of doubtful value.

*Medical Honors.*—Prof. James P. White, of this city, was recently elected a corresponding member of the Rhode Island State Medical Society. Such compliments to eminent members of the profession, contribute to good feeling, and professional fraternity. At the same time, Dr. Bigelow and Storer, of Boston, and one or two others, were also elected members.

*Statistics.*—We see daily the necessity of statistical facts in relation to disease. A friend recently complained to us, of the indefinite nature of his ideas of the treatment of dysentery. He had a patient ill with that disease, and he had given calomel, opium, astringents, cold and warm injections, cathartics, and sundry other remedies, at different times. All of these had been given without any very definite idea as to the effect to be produced. The patient was convalescent, and satisfied with his doctor, but the doctor himself was unable to assign to any one of his drugs, a positive share in the cure. And this is a fair statement of the treatment of very many cases of disease. It does not imply ignorance, or want of skill in the practitioner. It merely indicates a wide hiatus in the medical knowledge of all physicians. We have a good measure of skill already, but before us lies a field for research almost illimitable. Nothing short of full statistical accounts of diseases, their symptoms and treatment, can remedy this evil. A proper knowledge of our deficiencies is necessary to repairing them; but we would not go to the other extreme, and say, that for the want of this full information we are unable to combat disease with a good measure of succcss. We no not believe that.

"A *little* knowledge is a dangerous thing." It is a good thing, but the more of it the better. A paragraph is going the rounds of the secular papers, ascribed to the editor of the Medico-Chirurgical Review, in which he is represented as saying, that if all knowledge of drugs, and all doctors, were swept out of existence, there would be less disease and fewer deaths than now. This, if true, is an instance of that infidel spirit which is prevalent among the profession. But no such statement will bear the test of statistics. The mortality of the city of London, as shown by the mortuary tables, is less in proportion to the population than ever before; and the actual average of human life has been sensibly lengthened in the last two hundred years. All insurance tables also go to prove this fact. Now this increase of average human life has occurred in spite of the opposing influence of a higher civilization, and increased causes of disease. Greater skill in the management of disease is one cause, vaccination and sanitary reform are others; and it should be borne in mind, that for all these blessings, the world is indebted to the profession of medicine. With a self-abnegation displayed nowhere else, our calling has ever devoted itself to limiting its own income. Medical science prevents as much disease as it cures. It is ever foremost in sanitary reform. Sewerage, ventilation, the proper regulation of indoor temperature, and all other means of prophylaxis against disease, are taught with an earnestness and sincerity on the part of medical men, which does them infinite credit. A money-loving public adopts slowly, and thanklessly, the sanitary suggestions of medical science, and had the public been as ready to receive, as the profession to offer such information, the world would have seen less suffering.

We have faith in physic yet. Minds of super-refinement may theorize — they may mistake feeble vision for blindness — they may, in their search for the exact, and unchangeable, in that which God has made uncertain, and fickle as the wind, turn disappointed to a stubborn unbelief, as do German philosophers, who reason and refine upon their creeds, until they at last deny all truth — but other minds will labor in the more tedious paths of inquiry and investigation, and the medical profession will not only continue to exist, but will grow into a firmer and more intelligent public confidence. Its present existence alone is an evidence of its necessity; and we do not believe that such assertions as that of the editor of the Review, will ever lead a sick man to die for want of medical assistance. The most obdurate railers against medical art, are ever the most submissive of patients. S. B. H.

